# An On-Demand TDMA Approach Optimized for Low-Latency IoT Applications

**DOI:** 10.3390/s22176461

**Published:** 2022-08-27

**Authors:** Antonios Prapas, Konstantinos F. Kantelis, Petros Nicopolitidis, Georgios I. Papadimitriou

**Affiliations:** Department of Informatics, Aristotle University of Thessaloniki, 54124 Thessaloniki, Greece

**Keywords:** Internet of Things, LoRa, on-demand TDMA

## Abstract

The never-ending evolution of the Internet of Things ecosystem is reshaping the arena of wireless communications and competing against conventional networking solutions in fields such as battery life, device and deployment cost, coverage, and support for an immense number of devices. Inspired by this phenomenon, this paper presents a novel Medium Access Control protocol utilizing long-range technology, based on a Time Division Multiple Access communication protocol variant, adjusted to make better use of each device’s hardware. Focusing on Low Power Wide Area Network applications, this implementation improves data latency and offers amplified performance due to better network awareness and dynamic time slot rescheduling. Various simulation scenarios were contrived to evaluate the protocol’s performance. The results instate the proposed algorithm as a promising access scheme for the IoT field.

## 1. Introduction

Recently, the Internet of Things (IoT) has drawn substantial attention from a research standpoint. In the upcoming years, more and more devices will be connected to the IoT, with the estimated number of connected devices according to [1] rising to a whopping 38.6 and 50 billion by 2025 and 2030, respectively. The future of the Internet will consist of heterogeneously connected devices. A new type of network, Low Power Wide Area Networks (LPWANs), have enabled the communication between electronic devices and sensors through the Internet to improve and facilitate everyday life. The infrastructure for such a network is very lightweight and its unique characteristics of high communication range and rapid deployment support plenty of smart and intelligent applications [2], such as smart cities, environmental metering, forest and sea status surveillance, healthcare, logistics, and so on.

A very important element of LPWANs, especially for this work, is long-range (LoRa) transmission. It is a very promising and innovative long-distance wireless data transmission technology operating on the unlicensed ISM frequency band. Due to LoRa’s unique characteristics, it allows the communication of thousands of devices, at distances ranging from a few kilometers inside the urban web to tens of kilometers outside it, using a single gateway [3]. Moreover, the cost of the required equipment is particularly low, thus making this technology suitable for large-scale IoT applications.

The network consists of two basic device types, the end device (ED) and the sink node (SN) or base station. The former is a simple device with sensors attached on it, gathering information, and transmitting them back to the base station. The latter is a device in charge of controlling the whole network. It sends information between the ED and a central network server. Additionally, it has the capability to simultaneously support thousands of EDs at the same time.

The proposed algorithm makes optimized use of the hardware that was used in the work of [4], adding backwards communication from the EDs with the cluster head (CH) in favor of a more efficient LoRa Medium Access Control (MAC) protocol. The latter is a unique type of device dividing the total number of EDs into smaller groups, named clusters, and making sure that all of them are synchronized concerning the time slot each one will transmit its data to the SN. At the beginning of each cycle, the SN requests the data from the EDs by sending a LoRa message to the CH. Upon this message reception, the latter broadcasts the calculated transmission program to initiate the data transmission from the EDs via the wake-up radio (WuR) message. As the CH knows the distance to the SN for all the EDs, it divides them into two groups: those that are closer to the SN and have a lower spreading factor (SF), and those that are further, which assigns them a higher SF. As a result, EDs with a lower SF will also have lesser latency, and EDs that have higher SF are guaranteed that their transmissions will have improved range and higher probability of successful reception. Furthermore, the proposed protocol achieves a higher degree of slot utilization compared to a classic TDMA, as EDs that do not have any data to report inform the cluster of this fact, and as a result the remaining EDs adaptively reschedule their transmission time for a faster cycle, less latency, and zero empty slots.

The remainder of this paper is organized as follows: Section 2 overviews the related research on this field; Section 3 offers insights pertaining to the network architecture used in this work; the operation of the proposed algorithm is analyzed in detail in Section 4; and simulation results are demonstrated in Section 5. Finally, Section 6 concludes the paper.

## 2. Related Work

As the number of devices in an LPWAN increases, the carrier-sensing mechanism becomes less effective at reliably detecting channel activity, negatively affecting network performance. Therefore, LPWAN technologies such as Long-Range Wide Area Networks (LoRaWANs), built on top of LoRa, have been based on the pure-ALOHA protocol for uplink communication. ALOHA is an asynchronous protocol where the EDs communicate when they have data ready to send, either scheduled or event-driven. It is simple, lacks synchronization between the devices, and has minimum communication overhead. All these characteristics fit perfectly with the sporadic communication demands of applications with low traffic. Plenty of studies have been conducted to analyze the viability of this standard in terms of latency, reliability, and throughput, with most of them concluding that although the protocol performs adequately under a light load, it suffers from uplink traffic congestion in cases of heavy traffic as the number of network devices increases [5,6,7,8]. Slotted-ALOHA, a variant of the pure-ALOHA protocol, has been proposed as an alternative to enhance the overall system performance.

A frequent tactic used to improve battery life for most IoT devices is to resort to duty-cycling mechanisms, where the device periodically wakes up from sleep mode to retrieve new data. Although this approach allows for great power savings, it has its limitations. As the device wakes up based on a time schedule, it means that in some cases it will wake up even if there are no data for transmission, thus wasting energy and time. Additionally, if long sleep intervals are being used, then data latency increases. Trying to eliminate the above weaknesses, Piyare et al. [4] suggested an ingenious receiver-initiated On-Demand Time Division Multiple Access (TDMA) communication protocol for IoT applications to boost both the latency and the energy efficiency of standard LoRa architectures. Furthermore, ultra-low power WuRs, which are capable of continuously listening to the wireless channel and activate the system when a specific signal referred to as the wake-up beacon (WuB) is detected, are introduced in this work. WuR is the ideal solution for removing limitations such as long sleep intervals or idle listening posed by duty cycles, which lead to increased delay and energy expenditure, respectively.

Considering the aforementioned remarks, in this work, a new communication scheme is proposed for networks using the LoRa protocol, based on the standard TDMA approach. While keeping the collision-free characteristic of the TDMA protocol along with its simplicity, this work focuses on the node’s ability to communicate if it does not have a packet for transmission. Nodes continue to be synchronized based on the WuR [4]. In addition, after a time slot assignment to every node, whenever a node does not have data to report, it sends a LoRa message to the SH, and the time slot scheduling is reformed for the remaining nodes.

The proposed algorithm combines long-range communication with ultra-low power WuRs, achieving lower communication latency in heterogeneous long/short range networks when compared to typical TDMA implementations from the literature. With respect to [4] implementation, the main objective of this work is to further reduce the mean data latency, aiming at applications that have limited time response frames such as utility networks (water, electricity, and gas) which will greatly benefit from the aforementioned characteristics in case of an emergency such as water and gas leakages in addition to electrical network failures and many more. By giving the EDs the ability to communicate with the CH if they do not have a packet, the system reforms the time slots for the remaining EDs and saves time that otherwise would have been wasted. In the literature, several other architectures were already proposed (e.g., Industrial LoRa, RT-LoRa, RS-LoRa) where the beacon transmitted by the SN is used by the ED to dynamically (and automatically) choose the correct SF as a function of the distance from the SN [9,10,11]. A comparison of the proposed protocol with existing solutions is shown in Table 1. It should be mentioned that while the on-demand solution as presented herein does not use channel selection but only SF selection, an updated version for channel and SF selection is based primarily in a simple amendment of the existing WuR message used to inform about the SF. Utilizing additional bits in the aforementioned message, the system could exploit higher number of concurrent transmissions, serving a higher number of nodes at the same time.

## 3. Network Architecture

**Figure 1:**
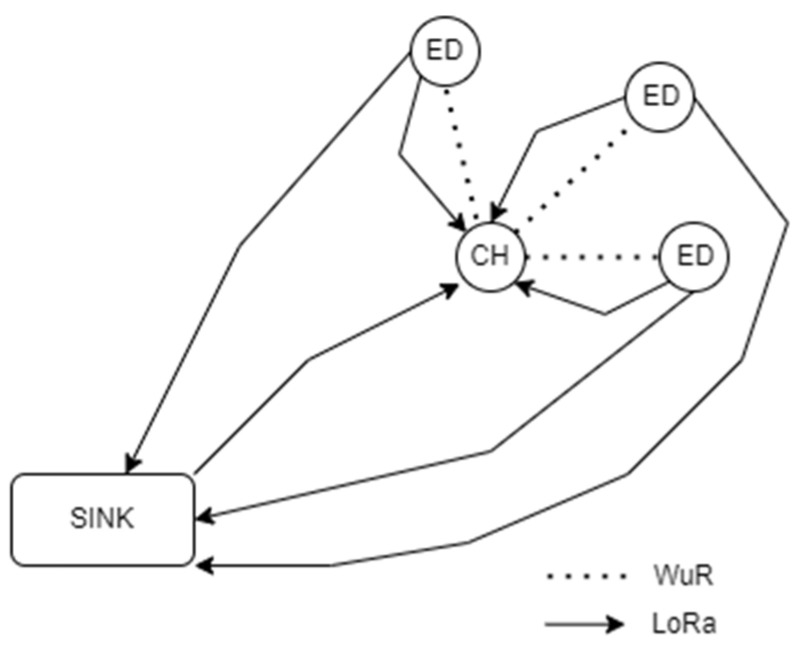
Network architecture.

This section describes in detail the architecture of the network that was adopted, as shown in Figure 1. To begin with, we are referring to a heterogeneous network consisting of three different types of nodes: the EDs, the CH, and the SN. The SN is the most important type, being unique in the network. It is the node where all the data are being gathered. The EDs are nodes equipped with different kinds of sensors that collect data and transmit them to the SN, and lastly the CH is the means of communication between the EDs and the SN. In the network, the EDs are split into one or more clusters, with every one of them having its own CH. Both CHs and EDs are equipped with a LoRa and a WuR transceiver [12,13,14,15]. Contrariwise, the SN is equipped only with a LoRa transceiver.

As a way to improve energy efficiency, reduce data latency and crashes, and integrate with IoT scalability, in this work we opted for the use of an ultra-low power wake-up radio transceiver. These devices (which mostly utilize the band below 1 KHz) can monitor the channel in a continuous mode, consume microwatts of power for addressing and wake-up operations of the major radio system. Exploiting the capabilities of these devices, we avoided false wake-ups, scheduled the main operation of the LoRa transceiver, and organized the nodes according to the proposed algorithm (vide infra). This type of communication is based solely on the activation of the system via interruptions whenever a specific signal referred to as a wake-up beacon is detected from the wake-up transmitter (WuTX). As a result, since the WuR can always be on, each node is additionally equipped with such a device and can operate in a purely asynchronous manner, activating the main radio on demand, without requiring continuous transmission. Upon reception of the WuB signal from the sender node, it immediately activates the node’s LoRa transceiver so as to transmit its available data via the LoRa-reserved spectrum. 

Since the WuR transceiver has a very low power consumption, it does not require continuous transmission, with direct effect on network life, as shown in Section 5. To allow for a power-efficient receiver implementation, the data are sent using on–off keying (OOK) modulation. The SN uses LoRa to send commands to the CHs $\left({\mathrm{SN}}_{\mathrm{LoRa}}\to \mathrm{CH}\right)$ and receive data from the EDs $\left({\mathrm{ED}}_{\mathrm{LoRa}}\to \mathrm{SN}\right)$. The CHs use a WuR message to synchronize every ED in the cluster and inform them of their time slot start $\left({\mathrm{CH}}_{\mathrm{WuR}}\to \mathrm{ED}\right)$. Additionally, they use LoRa to receive a flag signal from an ED when it does not have a packet to transmit on the current cycle $\left({\mathrm{ED}}_{\mathrm{LoRa}}\to \mathrm{CH}\right)$. Then, the CH resends a WuR signal to update all the still-waiting EDs of their new time slot start.

## 4. Operation of Proposed Approach

In the TDMA implementation by Piyare et al. in [4], they consider 9 end nodes (1–9), and every ED works with the same SF without considering its distance from the SN. This results in a significant difference in mean data latency, especially in cases where the larger SFs are being used for all EDs instead of a smaller one for some of the EDs. Moreover, for every ED a specific time slot is allocated to transmit its data over LoRa to the SN. The SN sends a message to the CH over LoRa, and then the latter sends a signal over WuR to all EDs and calculates the start of each time slot from the *WuBArrivalTime* as:

(1) $$T_{NextSlot}=WuBArrivalTime+\left(ToA_{pkt}+G_{t}\right)\cdot N_{id}$$

This results in significant delays in scenarios where not every ED has a packet to transmit, because the time slots cannot be updated dynamically, and the network waits for an empty transmission slot. The slot size is determined by computing the time-on-air ($ToA_{pkt}$) using the above equation (see Appendix A) for the LoRa data packet depending on the payload size, with a pre-defined guard time ($G_{t}$) of 6 ms, and $N_{id}$, which is the ID of each ED. The guard time guarantees that the window is large enough for the transmission and compensates for clock drift, which may be detrimental with an increasing number of EDs. Finally, the EDs start transmitting the data packets to the SN over the LoRa module, according to the slot schedule, as illustrated.

**Figure 2:**
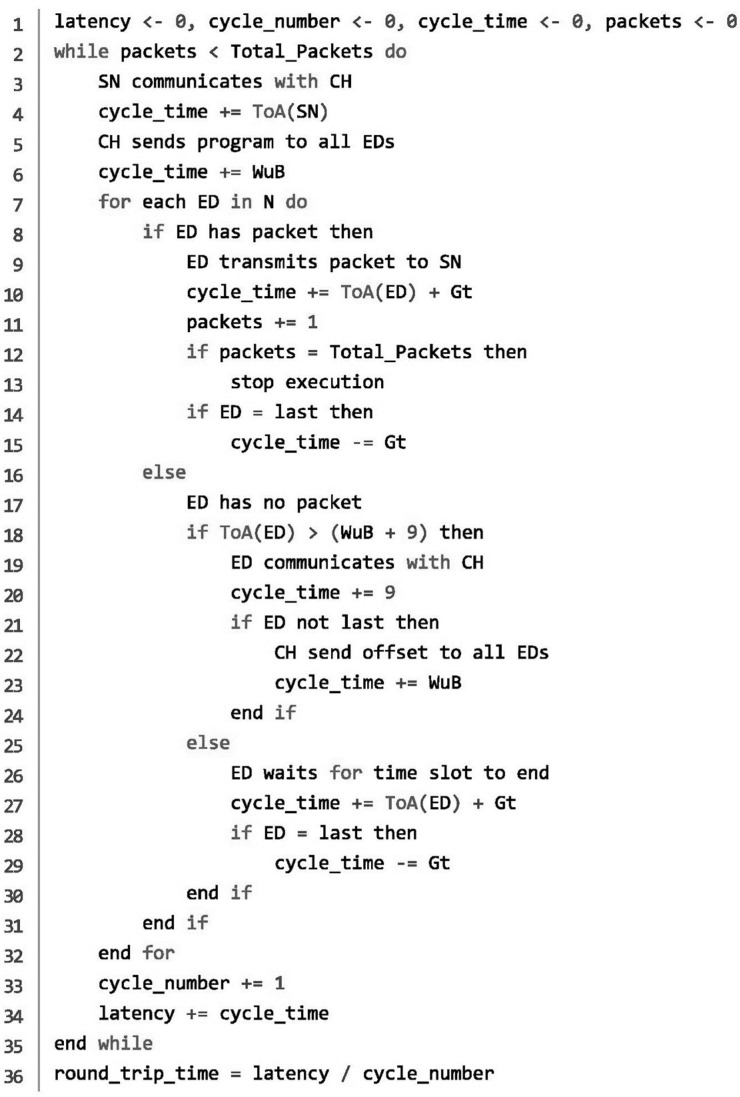
Distance-dependent TDMA algorithm.

**Figure 3:**
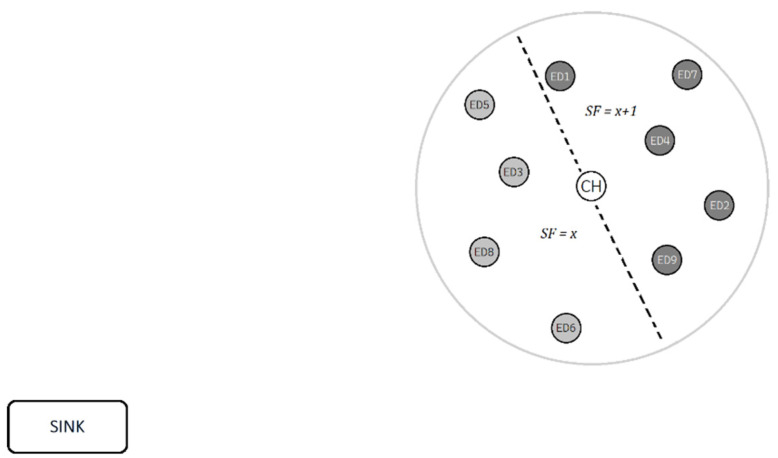
End devices using different spreading factors for communication with sink.

Firstly, the proposed algorithm (Figure 2) is aware in advance of the distance of every one of the 9 EDs and the CH from the SN, so it can use the most efficient SF possible (Table 2). We consider that in every scenario there will be at best two different SFs. That is true because we are aware of the operational range of LoRa being about 20 km. If we divide the LoRa range in six discrete SF zones (from 7 to 12), every zone has a radius larger than the previous one by about 3.333 km, and considering the range of WuR, which is 3 km, we end up with two SF groups that all the EDs in a cluster belong to. The first group will have from none to all the EDs and will operate with SF=x, and the second group will have the rest of the EDs and will operate with SF=x+1 (Figure 3). With that change we manage a smaller mean data latency, especially with the larger SFs due to the difference in time-on-air (ToA).

Additionally, the algorithm can dynamically calculate and set new starting points for every ED’s time slot when one or more of them do not have a packet to transmit. The SN sends a LoRa signal to the CH, and then the latter sends a message over WuR an informing the EDs about their scheduled transmission slots. The aforementioned WuR message has a different structure from a typical LoRa message, and as referenced above, it utilizes different radio technology. Recent works in this field have shown that WuR transceivers have the additional ability to transfer useful payload apart from the addressing information [12,13,14]. At the beginning of each cycle, as the CH knows the exact position of each node, it calculates the appropriate SF that should be used for message transmission for each of them. Nodes that are closer to the SN are directed to use the lower SF and the ones that are further away from the SN will use the higher one. Under this scheme, for every geographical configuration of nodes around the CH (balanced or not), the system can take advantage of the shorter ToA for the lower SF and the increased reliability for higher SF in relation to the distance. In order to achieve this, bit-stream coding is used in a dual-mix mode (as string and as binary number), to inform the nodes about their initial transmission time schedule and to alert the existing nodes of any empty transmission slots reported from the nodes.

**Figure 4:**
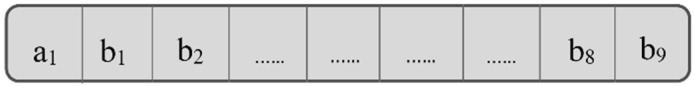
Wake-up radio message structure (additional part).

**Figure 5:**
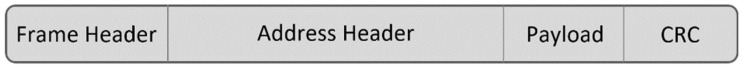
Wake-up radio addressing message packet structure.

Upon reception of this 10bit stream part (shown in Figure 4, which is additional to a typical WuR addressing message (shown in Figure 5), each node is able to calculate its transmission time slot by the following scheme:

- The first bit (a_1_) is used to distinguish the initial transmission program from any additional corrective message that informs the nodes about an unused transmission slot. The code for the initial schedule is translated as 1, while 0 means that the following bits are decoded as a binary number, standing for the node that will not use its time slot to transmit any data;
- The other 9 bits are used from the nodes, in the case of the initial program, to decode their transmission schedule. For each of the following 9 bits, each node sets its SF according to the appropriate bit. For example, ED_1_ uses the b_1_ bit, while node 9 uses the b_9_. A bit value of 1 means the node should use the higher of the two SFs while bit value of 0 indicates the use of the lower SF in each cycle;
- Whenever the first bit (a_1_) is equal to 0, the following bits are translated as a 9-bit binary number. The useful range for this number is from 1 to 8, as it stands for the node ID that will not transmit during its assigned time slot (there is no need to inform about the 9th node). Upon reception, each node adapts its transmission schedule and transmits its data one time slot sooner.

The above scheme ensures that each node is aware of the transmission schedule of all the other nodes in the cluster. Initially, each node calculates its time slot, also knowing the schedule of all the other nodes. Consequently, when a node sends a LoRa message to inform the CH with SF=7 that it will not make use of its assigned slot, the CH broadcasts a WuB message containing the ID of the node that will not report data. This message is transmitted via the WuR circuitry and being a broadcast message, it is ensured that all the nodes will receive it at the same time. Therefore, each node, knowing the reported node’s *SF*, reschedules its time slot. Correspondingly, the system utilizes all the available time slots to report the data, having direct results in network performance. Using a direct relation from the addressing of the WuB used in [4], the additional 10 bits that are used from the proposed protocol result in an additional 9.41 ms (17 ms + 9.41 ms = 26.41 ms) time as the total size is 3 bytes and an additional 2 bits (2 bytes used for addressing and 1 byte plus 2 additional bits for the enhanced WuB message). However, that process is activated only when the EDs use SFs>9, because otherwise the duration of the ED’s time slots is shorter than the update period. So, in that situation the network does not make any changes and just operates as the one in [4].

## 5. Simulation Results

**Figure 6:**
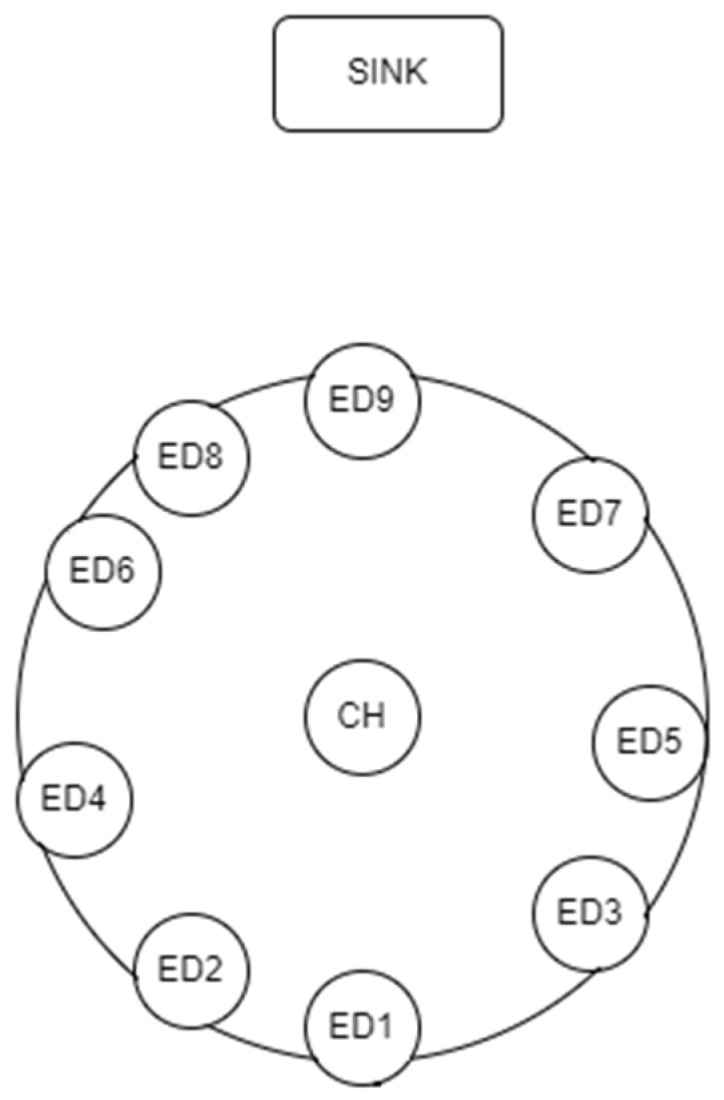
Testing network.

This section presents the simulation results for the Distance-Dependent TDMA protocol, to assess its improvement in mean data latency over the implementation of [4]. The parameters for the simulation runs are presented in Table 3. There was some energy efficiency testing as well. It should be noted here that the mean data latency pertains to the round-trip time latency, which in turn is computed as the time difference between the SN transmitting the initial LoRa message to the CH and receiving all LoRa messages from the EDs. The energy measurements are based on [4] as well, using a 1200 mAh lithium polymer battery at 3.3 V and the power consumption numbers that Piyare uses. Both protocols have been implemented using Python 3.8 (Python Software Foundation, Wilmington, DE, USA) on a Jupyter Notebook (4.9.1, Professor Fernando Pérez, UC Berkeley, CA, USA) running Windows 11 with AMD Ryzen 5 5600 H. Two network architectures were simulated at four different network load situations. The network shape in Figure 6 is mutual for both networks and only the distances of the nodes vary (Table 4 and Table 5). Both networks were tested with packets following: (i) Uniform distribution, (ii) Normal distribution, (iii) Binomial distribution and (iv) Poisson distribution.

The uniform distribution is the one that Piyare et al. use in [4], and it basically means that every ED has a packet for the SN on every cycle. This is a very unrealistic scenario, so more lifelike distributions were tested. For the normal distribution the parameters used were loc=ED2 and scale=1.5, for the binomial distribution n=ED+1 and p=0.65 was used, and for the Poisson distribution lam=ED3 (see Appendix B). These distributions were used to set how many EDs have a packet to transmit and feeds them with messages one at a time (EDs do not have any buffer as in the referenced system).

**Figure 7:**
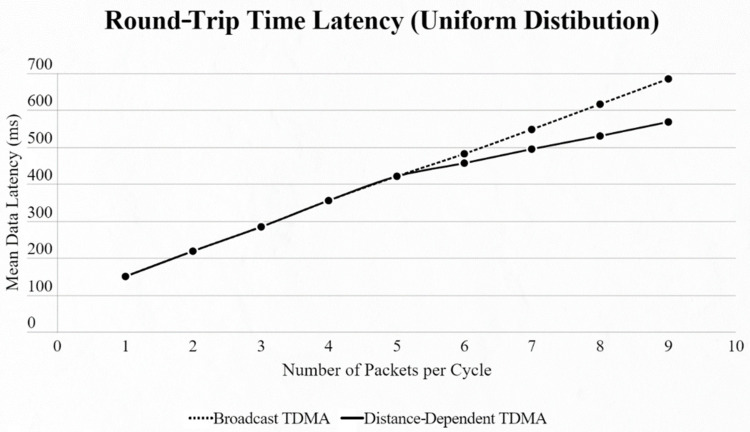
First network mean data latency using uniform distribution.

**Figure 8:**
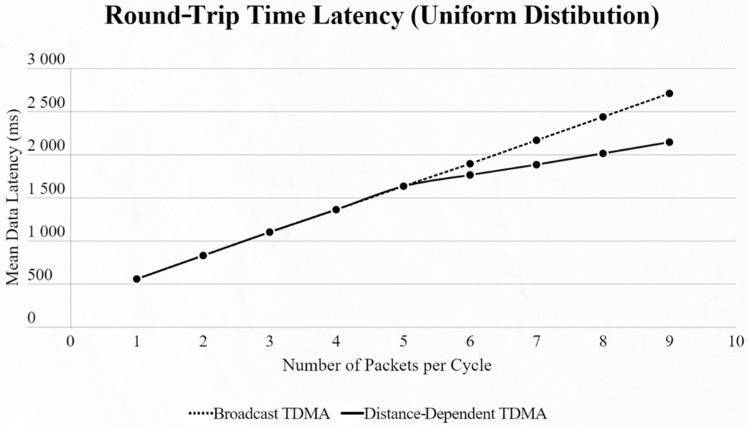
Second network mean data latency using uniform distribution.

For both networks, the results were basically the same, but in different scales. When using the uniform distribution and changing the number of EDs in the network, we observed that until we reached 5 EDs the two approaches had the exact same results. From that point we faced lower data latency using our approach, due to the use of a smaller SF exploiting the ability of our algorithm to find the most suitable SF for every ED based on its distance from the SN (Figure 7 and Figure 8).

**Figure 9:**
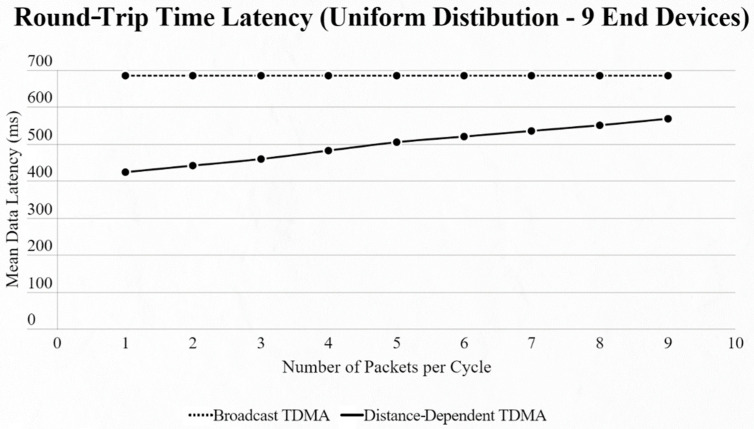
First network mean data latency using uniform distribution under variable packet number per cycle with 9 end devices.

**Figure 10:**
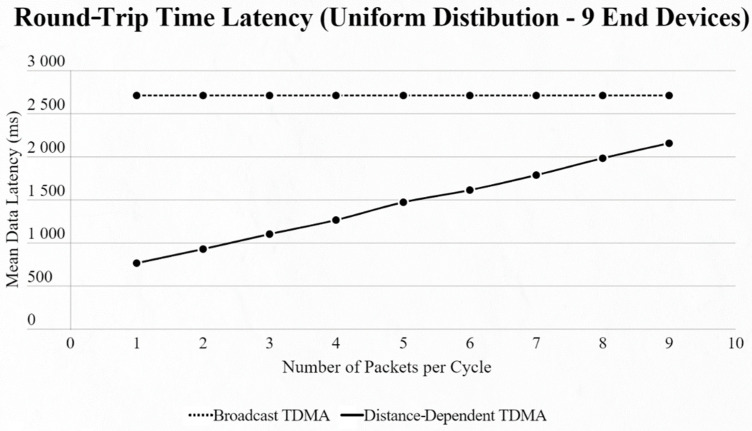
Second network mean data latency using uniform distribution under variable packet number per cycle with 9 end devices.

On the other hand, if we use the uniform distribution again but with a fixed number of 9 EDs in the network and our testing variable is the number of packets transmitted per cycle, we notice that [4] has a steady mean data latency regardless of the changes, as the algorithm cannot sense if an ED has a packet or not, in contrast to our approach, which has a linear increase thanks to ED and CH communication (Figure 9 and Figure 10).

**Figure 11:**
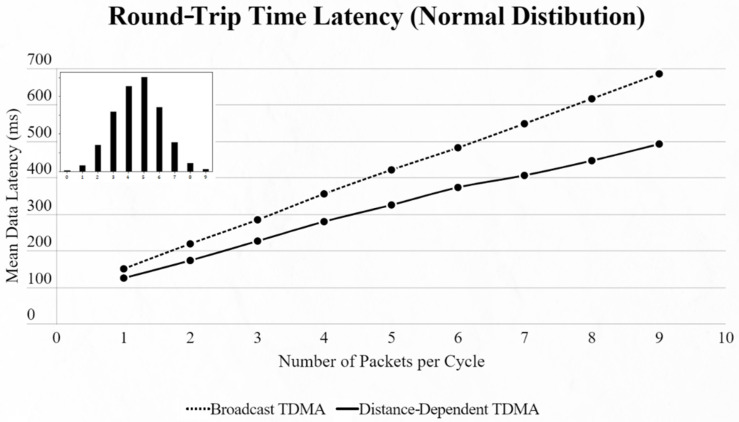
First network mean data latency using normal distribution.

**Figure 12:**
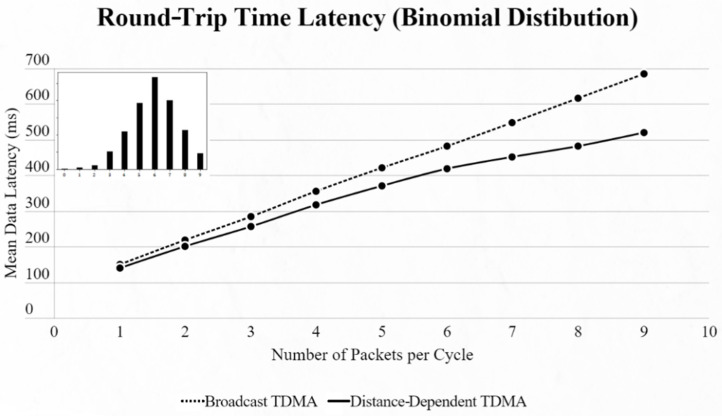
First network mean data latency using binomial distribution.

**Figure 13:**
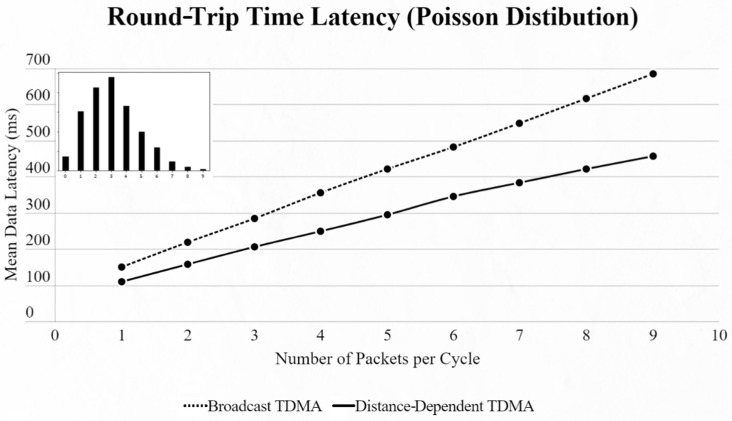
First network mean data latency using Poisson distribution.

**Figure 14:**
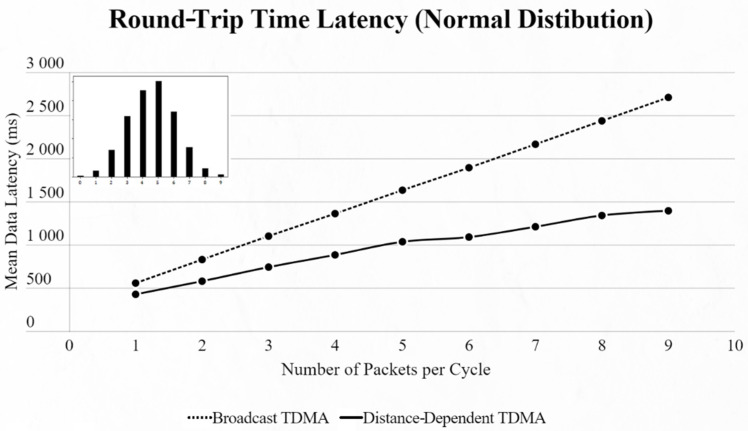
Second network mean data latency using normal distribution.

**Figure 15:**
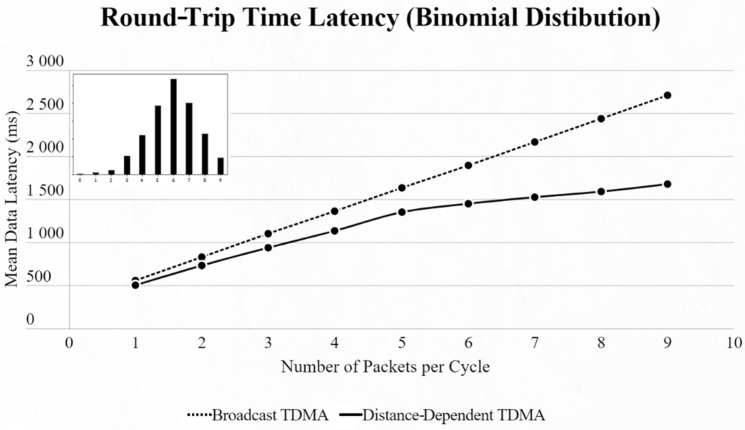
Second network mean data latency using binomial distribution.

**Figure 16:**
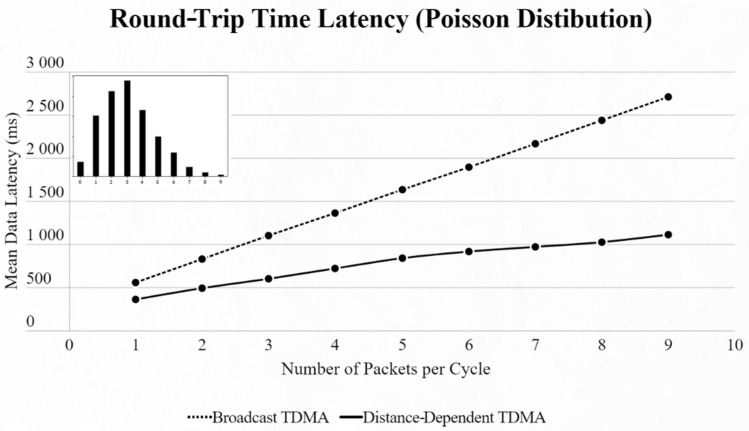
Second network mean data latency using Poisson distribution.

By using the other three types of distribution (normal, binomial and Poisson), we can clearly distinguish the advantages of the proposed protocol. In every scenario, the mean data latency is lower for any given number of EDs, especially after more than 5 EDs are added to the network where the selection of the proper SF takes place. The biggest difference is shown using the Poisson distribution, as the mean number of packets on every cycle is about 3, followed by the normal distribution which has a 4.5 packet-per-cycle rate and lastly the binomial distribution with 6.5 packets per cycle. On the top-left corner of each time graph we can discern how many cycles with each number of packets have been assigned for every distribution in a 9-ED network (Figure 11, Figure 12, Figure 13, Figure 14, Figure 15 and Figure 16).

Looking at the mean data latency of the two tested networks side by side, we notice that the gap between [4] and our approach is greater in the second network, which uses bigger SFs. This is because the ToA does not increase linearly with every SF increment:

$$\left\{\begin{array}{c} D_{9,10}=ToA_{10}-ToA_{9}=62-31=31\;\mathrm{ms}\;\\ D_{11,12}=ToA_{12}-ToA_{11}=264-124=140\;\mathrm{ms} \end{array}\right.$$

**Figure 17:**
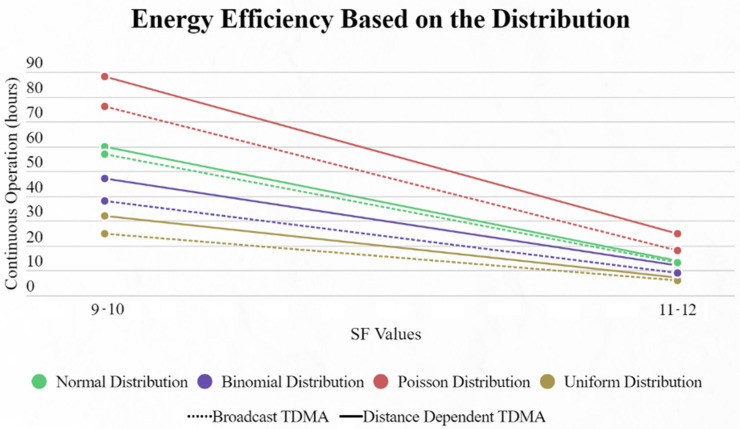
Energy endurance for broadcast TDMA and distance-dependent TDMA based on the distribution used.

Lastly, we compared the energy efficiency of this approach over Piyare’s. The results presented below were exported using a unique method. We used the battery capacity of 1200 mAh operating at 3.3 V, exactly like Piyare. That gives us a 3.96 Wh battery. In the testing method we took the average power consumption of all 9 EDs of the network and we constantly ran simulations. There were no pauses, meaning that at the end of each cycle the SN immediately started a new one, and we measured how many hours the average ED would last for every distribution until its battery ran out. We can clearly distinguish (Figure 17) that in every scenario our protocol manages to outperform Piyare’s approach concerning the endurance of the network in a continuous mode. If we consider that each cycle will happen with an inter-packet interval of 10 s, as Piyare used for his simulations, the improvement is significant. 

## 6. Conclusions

In this work, some simple yet effective modifications to Piyare’s approach were introduced, improving the network’s architecture in real-life conditions where not every ED has a packet for the SN in every cycle. Moreover, since the protocol has a smaller cycle time, it has the means to offer a boost in the energy efficiency of the ED. This boost originates from the fact that EDs stay awake for less time and improvements are purely software-based, with no requirement for extra hardware. The performance characteristics of the model are amplified using higher SFs and cycles where only a few EDs have a packet to transmit. Nevertheless, for SFs≤9 the protocol works exactly like the one referenced from Piyare et al., and has improvements in cases where EDs operate under various SFs inside the same cluster.

## Figures and Tables

**Table 1:** Main characteristics of LoRaWAN, industrial LoRa, RT-LoRa, RS-LoRa and our approach.

	LoRaWAN	Industrial LoRa	RT-LoRa	RS-LoRa	Proposed Approach
**Topology**	Star	Star	Star	Star	Star
**Synchronization**	Not supported	Beacon-based	Multi-beacon based	Beacon-based	WuR Messages
**Transmission of real-time periodic flows**	Not supported	Supported	Supported	Not supported	Not supported
**Smart selection of Spreading Factors**	Supported through ADR	Not supported	Supported through multiple beacons	Supported through beacons	WuR Messages
**MAC strategy for aperiodic transmission**	Pure ALOHA	Pure ALOHA	Slotted ALOHA	Pure ALOHA	Pure ALOHA
**MAC strategy for periodic transmission**	Pure ALOHA	Multi-CH and Multi-SF TDMA	Multi-CH and Multi-SF TDMA	Multi-CH and Multi-SF TDMA	Multi-SF TDMA
**QoS classes**	Downlink only (3 device classes provided)	Not supported	Uplink only (3 device classes provided)	Not supported	Not supported
**Support for retransmission**	Uplink only	Not supported	Not supported	Uplink only	Not supported
**Frequency rotation**	Pseudo-random channel hopping	Not supported	Supported	Not supported	Not supported

**Table 2:** Time-on-air for every SF.

	SF					
	7	8	9	10	11	12
**DE**	0	0	0	0	0	1
**CR**	1	1	1	1	1	2
**Time-on-air**	9	18	31	62	124	264

**Table 3:** LoRa radio settings.

LoRa Radio Setting	SET 1	SET 2	SET 3	SET 4	SET 5	SET 6
Spreading Factor	12	11	10	9	8	7
Coding Rate	4/6	4/5	4/5	4/5	4/5	4/5
Bandwidth (kHz)	500	500	500	500	500	500
Data Rate (kb/s)	0.976	2.14	3.9	7.03	12.5	21.87
Transmission Power	10	10	10	10	10	10
Payload (B)	8	8	8	8	8	8
Preamble Length (symbols)	8	8	8	8	8	8
Carrier Frequency (MHz)	868	868	868	868	868	868
Time-on-air	264	124	62	31	18	9

**Table 4:** First network node distances.

		SF (ToA)	
Route	Distance (m)	Broadcast TDMA	Distance Dependent TDMA
**S → CH**	10.000	10 (62 ms)	10 (62 ms)
**ED1 → S**	13.000	10 (62 ms)	10 (62 ms)
**ED2 → S**	12.500	10 (62 ms)	10 (62 ms)
**ED3 → S**	12.000	10 (62 ms)	10 (62 ms)
**ED4 → S**	11.000	10 (62 ms)	10 (62 ms)
**ED5 → S**	10.500	10 (62 ms)	10 (62 ms)
**ED6 → S**	9.000	10 (62 ms)	9 (31 ms)
**ED7 → S**	8.000	10 (62 ms)	9 (31 ms)
**ED8 → S**	7.500	10 (62 ms)	9 (31 ms)
**ED9 → S**	7.000	10 (62 ms)	9 (31 ms)

**Table 5:** Second network node distances.

		SF (ToA)	
Route	Distance (m)	Broadcast TDMA	Distance Dependent TDMA
**S → CH**	17.000	12 (264 ms)	12 (264 ms)
**ED1 → S**	20.000	12 (264 ms)	12 (264 ms)
**ED2 → S**	19.500	12 (264 ms)	12 (264 ms)
**ED3 → S**	19.000	12 (264 ms)	12 (264 ms)
**ED4 → S**	18.000	12 (264 ms)	12 (264 ms)
**ED5 → S**	17.300	12 (264 ms)	12 (264 ms)
**ED6 → S**	16.000	12 (264 ms)	11 (124 ms)
**ED7 → S**	15.000	12 (264 ms)	11 (124 ms)
**ED8 → S**	14.500	12 (264 ms)	11 (124 ms)
**ED9 → S**	14.000	12 (264 ms)	11 (124 ms)

## Appendix A

For a given combination of LoRa setting, spreading factor, coding rate, and bandwidth, the total time-on-air for a given payload size is determined as follows:

$$ToA_{pkt}=\left[\left(n_{pre}+4.25\right)\cdot \frac{1}{R_{sym}}\right]+\left[8+max\left(ceil\left[\frac{8PL-4SF+28+16CR-20H}{4\left(SF-2DE\right)}\right]\cdot \left(CR+4\right),\;0\right)\cdot \frac{1}{R_{sym}}\right]$$

where:

- $n_{pre}$ is the programmed preamble length;
- $R_{sym}$ is the symbol rate;
- PL is the payload length in bytes (1–255);
- SF is the LoRa spreading factor (6–12);
- H is the packet header, zero when the header is enabled and one when no header is present;
- DE is the data rate optimizer, one when enabled, zero otherwise;
- CR is the LoRa coding rate [1: 4/5, 2: 4/6, 3: 4/7, 4: 4/8].

## Appendix B

- Normal Distribution: $f\left(x\right)=\frac{1}{scale\;\cdot \;\sqrt{2\pi }}e^{-\frac{1}{2}{\left(\frac{x-loc}{scale}\right)}^{2}}$;
- Binomial Distribution: $f\left(k\right)=\left(\begin{array}{c} n\\ k \end{array}\right)\cdot p^{k}\cdot {\left(1-p\right)}^{n-k}$, for $k\in \left\{0,\;1,\;2,\;\ldots ,\;n\;\right\}$;
- Poisson Distribution: $f\left(k;lam\right)=\frac{lam^{k}\;\cdot \;e^{-lam}}{k!}$.
